# MECHANISMS IN ENDOCRINOLOGY: The sexually dimorphic role of androgens in human metabolic disease

**DOI:** 10.1530/EJE-17-0124

**Published:** 2017-05-03

**Authors:** Lina Schiffer, Punith Kempegowda, Wiebke Arlt, Michael W O’Reilly

**Affiliations:** 1 Institute of Metabolism and Systems ResearchUniversity of Birmingham, Edgbaston, Birmingham, UK; 2 Centre for EndocrinologyDiabetes and Metabolism, Birmingham Health Partners, University Hospitals Birmingham NHS Foundation Trust, Edgbaston, Birmingham, UK

## Abstract

Female androgen excess and male androgen deficiency manifest with an overlapping adverse metabolic phenotype, including abdominal obesity, insulin resistance, type 2 diabetes mellitus, non-alcoholic fatty liver disease and an increased risk of cardiovascular disease. Here, we review the impact of androgens on metabolic target tissues in an attempt to unravel the complex mechanistic links with metabolic dysfunction; we also evaluate clinical studies examining the associations between metabolic disease and disorders of androgen metabolism in men and women. We conceptualise that an equilibrium between androgen effects on adipose tissue and skeletal muscle underpins the metabolic phenotype observed in female androgen excess and male androgen deficiency. Androgens induce adipose tissue dysfunction, with effects on lipid metabolism, insulin resistance and fat mass expansion, while anabolic effects on skeletal muscle may confer metabolic benefits. We hypothesise that serum androgen concentrations observed in female androgen excess and male hypogonadism are metabolically disadvantageous, promoting adipose and liver lipid accumulation, central fat mass expansion and insulin resistance.

## Introduction

**Figure 1:**
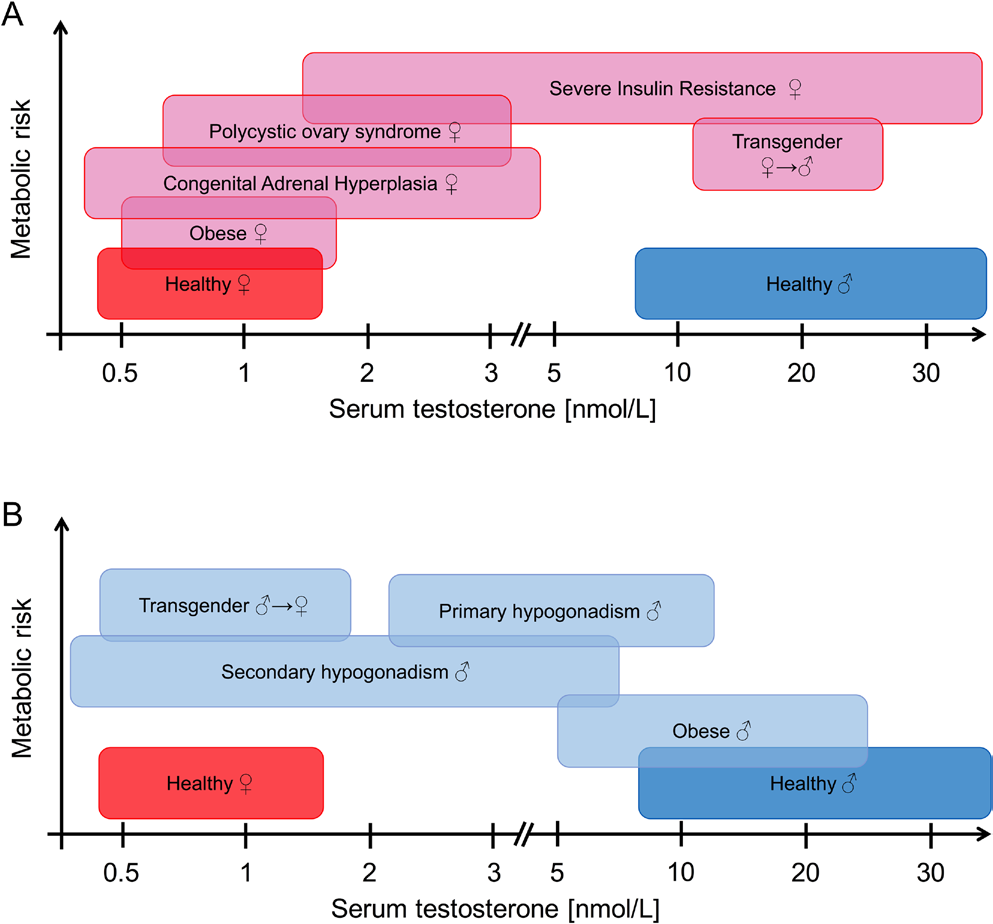
Sexually dimorphic associations between circulating testosterone levels and increasing metabolic risk. The estimated metabolic risk for different populations suffering from femal androgen excess (Panel A) or male androgen deficiency (Panel B) is shown in relation to testosterone levels. Serum testosterone concentrations of women with androgen excess and men with androgen deficiency overlap and are associated with severe adverse metabolic consequences leading to the concept of the ‘metabolic valley of death’ as a metabolically adverse window of circulating androgen concentrations. Approximate hormone ranges are taken from recent publications using mass spectrometry-based quantification: Healthy women vs PCOS women (200), obese women (30), women with CAH on standard glucocorticoid replacement therapy (201), healthy and obese men (202), men with primary hypogonadism due to Klinefelter syndrome not receiving testosterone supplementation (203), men with secondary hypogonadism due to idiopathic hypogonadotropic hypogonadism and hypopituitarism (204), as well as male-to-female and female-to-male transgender patients (70). No information about the method used to determine serum testosterone in women with type A form of severe insulin resistance was available, but values are included for completeness (205).

Disturbances in androgen metabolism secondary to gonadal, adrenal or hypothalamic–pituitary disease lead to alterations of circulating androgen concentrations, and result in reproductive and metabolic complications. In women, polycystic ovary syndrome (PCOS), a triad of ovulatory dysfunction, polycystic ovarian morphology and androgen excess (AE), represents the most common endocrine disorder (1). In men, disturbances of gonadal function most commonly result in hypogonadism and consequent androgen deficiency (AD), which can be inherited or acquired by disease, obesity, medications or the ageing process (2). Interestingly, female AE and male AD are associated with a similar adverse metabolic phenotype, including obesity, insulin resistance (IR), an increased prevalence of type 2 diabetes mellitus (T2DM), non-alcoholic fatty liver disease (NAFLD), cardiovascular disease (CVD) and even premature mortality (3, 4, 5, 6, 7, 8). This highlights a sexual dimorphism in the relationship between androgens and metabolism. As serum testosterone (T) concentrations in female AE and male AD may overlap, Escobar-Morreale *et al*. have proposed the concept of a metabolically adverse window of circulating androgen concentrations that are associated with deleterious metabolic consequences (9), or a ‘metabolic valley of death’ (Fig. 1). However, the cellular and systemic mechanisms underpinning these phenomena are poorly understood. In this article, we will discuss disorders of AE in women and AD in men, examine the role of androgens in the function of metabolic target tissues, and compare phenotype and consequences of metabolic dysfunction in the context of AE and AD.

## Pre-receptor androgen synthesis and metabolism

Androgens are 19-carbon (C19) steroid hormones produced by the adrenal gland and gonads in both men and women; they derive from C21 precursor steroids and can be converted to C18 steroids, the oestrogens. The androgen precursor steroids dehydroepiandrosterone (DHEA) and androstenedione (A4) are secreted mainly by the adrenal glands in both sexes, and by the ovary in females. Active T is produced directly in testicular Leydig cells in men and ovarian theca cells in women, but may also be activated from precursors in peripheral tissues (10), and can be generated in small amounts by the adrenal gland (10). T can be converted downstream to the more potent androgen 5α-dihydrotestosterone (DHT) by 5α-reductase activity. T and DHT bind and activate the androgen receptor (AR), eliciting classic genomic androgen action.

**Figure 2:**
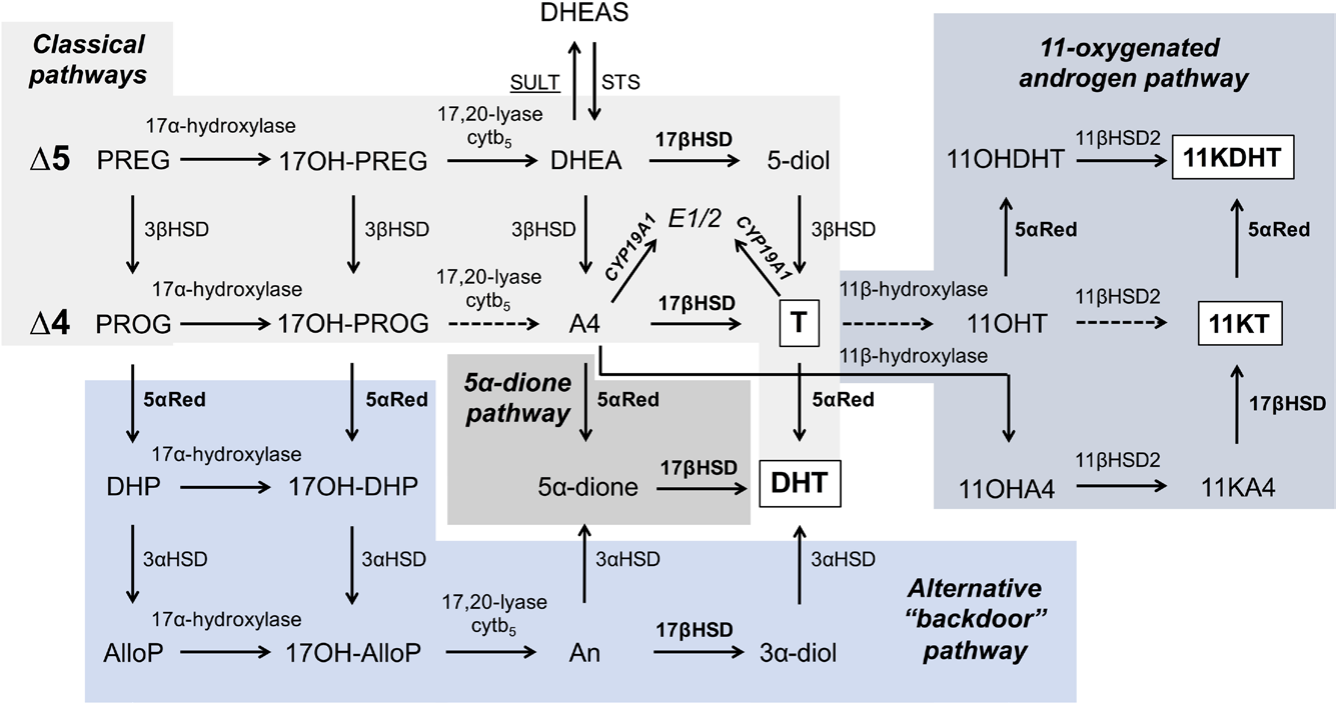
Overview of the human androgen biosynthesis pathways. Pregnenolone (PREG), produced by the side-chain cleavage of cholesterol, is the common precursor of all androgen biosynthesis pathways. The classical pathways, proceeding parallel for ∆5- and ∆4-precursors, lead to the formation of testosterone (T), which can be converted to dihydrotestosterone (DHT). The alternate 5α-dione pathway and ‘backdoor’ pathway directly synthesise DHT by-passing T. The 11-oxygenated androgen pathway converts androstenedione (A4) to 11β-hydroxyandrostenedione (11OHA4) by adrenal 11β-hydroxylase (CYP11B1) activity, generating the active androgens 11-keto-testosterone (11KT) and 11-keto-dihydrotestosterone (11KDHT). CYP17A1 capable of both 17α-hydroxylase and 17,20-lyase activity. All androgen receptor-transactivating androgens (T, DHT, 11KT and 11KDHT) are highlighted in bold and white boxes. Enzymes upregulated in PCOS contributing to local and systemic androgen excess (steroid 5α-reductase, 5αRed; 17β-hydroxysteroid dehydrogenase, 17βHSD) are highlighted in bold. Impaired activity of sulfotransferase 2A1 (SULT, underlined) due to mutations of the co-factor synthesising PAPS synthase 2 leads to a PCOS-like phenotype. Androstenedione and T can be converted to the oestrogens estrone (E1) and estradiol (E2), respectively, by aromatase (CYP19A1), whose activity possibly enhances androgen deficiency in obese men. Steroid abbreviations: 3α-diol, 5α-androstanediol; 5α-dione, 5α-androstanedione; 5-diol, androstene-diol; 11KA4, 11-keto-androstenedione; 11OHDHT, 11β-hydroxytestosterone; 17OH-AlloP, 17-hydroxyallopregnanolone; 17OH-DHP, 17-hydroxydihydroprogesterone; 17OH-PREG, 17-hydroxypregnenolone; 17OH-PROG, 17-hydroxyprogesterone; AlloP, allopregnanolone; An, androsterone; DHEA, dehydroepiandrosterone; DHEAS, dehydroepiandrosterone sulfate; DHP, 5α-dihydroprogestrone; PROG, progesterone. Enzyme abbreviations: STS, steroid sulfatase; 3β-HSD, 3β-hydroxysteroid dehydrogenase/∆4–5 isomerase; 11βHSD2, 11β-hydroxysteroid dehydrogenase type 2; cytb_5_, cytochrome b5.

Androgens can be synthesised from cholesterol via three interconnected pathways, which are schematically visualised in Fig. 2. The classical pathway produces T, which is activated to DHT in peripheral target tissues. There are several alternative pathways to DHT synthesis that bypass the classic synthesis pathway; the so-called backdoor pathway (11, 12, 13) and alternate 5α-dione pathway (14, 15) that directly synthesise DHT by-passing T. In healthy men, circulating T concentrations are approximately 10-fold higher than those observed in women (16). Besides *de novo* biosynthesis, active androgens can be synthesised from circulating androgen precursors in peripheral tissues expressing the required enzymes, thereby modulating local androgen exposure. In adipose tissue, A4 is converted to T by 17β-hydroxysteroid dehydrogenase type 5 (17β-HSD5), also called as aldoketoreductase type 1C3 (AKR1C3), and T may be further activated to DHT by the type 1 isoform of 5α-reductase (17).

Recently, it has been shown that steroids downstream of the major adrenal androgen precursor 11β-hydroxyandrostenedione (11OHA4), generated from A4 via the adrenal CYP11B1 enzyme (18), are active 11-oxygenated androgens (19). 11-keto-testosterone (11KT) and 11-keto-5α-dihydrotestosterone (11KDHT) (Fig. 2) have been shown to have the same AR activating potential as T and DHT, both with regard to affinity and transactivation potential (20), raising the possibility of an important role for these previously overlooked androgens in conveying biological androgen action (21). While all four agonists have comparable maximum transactivation potential for the AR, DHT and 11KDHT also have an AR affinity that is approximately one order of magnitude higher than the affinity of T and 11KT highlighting the importance of peripheral 5α-reductase activity for androgen action (21). Importantly, the circulating levels of 11KT have been shown to be approximately four times higher than those of T in healthy premenospausal women, which demonstrates the significant contribution of 11-oxygenated androgens not only to the androgen precursor pool, but also to the pool of circulating active androgens (22).

## Androgen excess in women and related metabolic consequences

### Polycystic ovary syndrome

PCOS is the most common cause of AE in women, affecting 5–10% of women of reproductive age (4, 23). PCOS is diagnosed according to the 2003 Rotterdam criteria (24), with two of the following three features required for diagnosis: ultrasound appearance of polycystic ovarian morphology (PCO), anovulation (AO) and AE. However, PCOS is also a major metabolic disorder, associated with IR, visceral adiposity and obesity, dyslipidaemia, NAFLD, CVD and potentially premature mortality (3, 4). PCOS-associated metabolic dysfunction is intimately linked with AE (25) (Fig. 1). Conventionally, circulating androgen burden has been typically evaluated by measuring serum T (25, 26), but recent work has defined A4 as a more sensitive marker for detecting PCOS-related AE, as well as demonstrating that integrated assessment of A4 and T is predictive of adverse metabolic risk (22, 27). Increased circulating concentrations of the DHEA sulfate ester DHEAS and 11OHA4, as well as active 11-oxygenated androgens, are indicative of AE of adrenal origin in PCOS. The latter was explored for the first time in a recent study, which demonstrated that more than half of the circulating androgen pool in a large cohort of PCOS women consisted of 11-oxygenated androgens (22), highlighting the significant adrenal contribution to PCOS-related AE. Of note, this increase in circulating 11-oxygenated androgens was similarly observed in obese and non-obese PCOS women, raising the question whether androgen excess precedes the development of metabolic complications.

In addition to systemic AE, tissue-specific androgen activation and its dysregulation contribute to local androgen burden. Systemic upregulation of 5α-reductase activity is observed in PCOS (28, 29, 30); resulting in enhanced activation of T to DHT; this phenomenon is already observed in daughters of PCOS women in early childhood (31). However, it is controversial whether daughters of PCOS women also develop a metabolic and biochemical phenotype and ovarian morphology characteristic of PCOS during puberty (32, 33). Overexpression of the steroidogenic enzyme AKR1C3 in PCOS adipose tissue is likely to contribute to tissue-specific AE, as this is the only enzyme expressed in adipose tissue that can locally generate T from A4 via its 17βHSD activity (34). AKR1C3 expression is increased in adipose tissue from patients with simple obesity and decreases with weight loss (34); furthermore, AKR1C3 expression in adipose tissue from PCOS patients is higher than in body mass index (BMI)-matched controls (35). Weight loss has been shown to represent an effective treatment to ameliorate PCOS-associated AE, ovulatory dysfunction and metabolic issues (36), further supporting an important role for adipose tissue as an organ of androgen generation in PCOS.

### Women with monogenic causes of androgen excess

The variants of congenital adrenal hyperplasia (CAH) represent a group of inborn disorders with autosomal recessive inheritance characterized by glucocorticoid deficiency and variable impact on mineralocorticoid and androgen secretion. Three CAH variants are associated with AE in affected women: 21-hydroxylase deficiency, 11β-hydroxylase deficiency and 3β-hydroxysteroid dehydrogenase type 2 deficiency. The most common defect is 21-hydroxylase deficiency, with a frequency of 1:16 000 in newborns (37, 38) and is the only enzyme deficiency frequently resulting in a non-classic CAH form with only mild glucocorticoid deficiency, but relevant AE (39, 40). As a consequence of the enzymatic block, precursor steroids are shunted down the pathways of androgen biosynthesis, which is further increased by enhanced hypothalamic–pituitary adrenal drive due to the loss of the negative feedback by cortisol (18, 41). While patients with major loss-of-function mutations usually present at birth or in early childhood, patients with mild mutations are often only diagnosed in early adulthood, as their glucocorticoid and mineralocorticoid secretion is sufficiently upheld by continuously increased ACTH stimulation of the adrenals, at the expense of AE. These patients usually do not present with outright virilisation, but generally with a PCOS phenotype in adolescence or early adulthood, including hirsutism, irregular periods and PCO appearance of the ovaries. In patients with non-classic CAH, an increased prevalence of obesity and insulin resistance has been reported (42, 43, 44), mirroring the adverse metabolic phenotype found in PCOS. As PCOS represents a diagnosis of exclusion and on average 2–3% of women presenting with a PCOS phenotype are identified as suffering from non-classic CAH (4), screening for CAH by baseline serum 17-hydroxyprogesterone is recommended in the work-up of PCOS.

Recently, another monogenic cause of AE, PAPSS2 deficiency (PAPSS2, 3′-phosphoadenosine 5′-phosphosulfate synthase 2), has been described to present with a PCOS-like phenotype (45). PAPS is the universal sulfate donor, generated by the two human PAPS synthase isoforms, and inactivating muations in PAPS synthase 2 have been shown to result in significantly impaired DHEA sulfotransferase (SULT2A1) activity (46). Consequently, fewer molecules of the androgen precursor DHEA are inactivated to DHEAS, resulting in increasing rates of conversion of DHEA towards T and DHT (Fig. 2). The first reported case, a homozygously affected young girl, presented with premature pubarche followed by irregular cycles and secondary amenorrhoea; investigations revealed AE with non-detectable serum DHEAS. Interestingly, her heterozygous mother, who harboured a major loss-of-function mutation on one allele, had presented with PCOS as a young woman (45). A further family affected by PAPSS2 deficiency was recently identified, and work-up revealed significant AE not only in the affected children but also in the heterozygous mother, co-incidentally again the carrier of a major loss-of-function mutation, with clinical manifestation as PCOS (47).

### Women with monogenic insulin resistance

Severe insulin resistance can develop independent of obesity as a consequence of monogenic gene defects impacting on insulin signalling or adipose tissue development. Defects in insulin signalling can be found at the level of the insulin receptor or in post-receptor signal transduction. Monogenic disorders may also cause severe obesity and consequent IR, or dysfunctional adipose tissue development resulting in congenital complete or partial lipodystrophy (48). Patients with IR due to monogenic lipodystrophy or insulin receptor (INSR) mutations present with AE, ovulatory dysfunction, PCO and acanthosis nigricans, usually in the absence of obesity. Compensatory hyperinsulinaemia may stimulate ovarian androgen biosynthesis by direct effects of insulin on theca and stromal cells (49), although other peripheral sources of insulin-stimulated androgen generation cannot be discounted. Monogenic INSR mutations may be suspected clinically in the setting of severe hyperinsulinaemia, which is accompanied by normal or elevated levels of leptin, adiponectin and SHBG, alongside a normal lipid profile and absence of hepatic steatosis (48).

## Androgen deficiency in men and related metabolic consequences

Male AD is a clinical syndrome arising from failure of testicular T production, in the context of primary testicular pathology or hypothalamic–pituitary disease (2). In adult men, it is diagnosed by the presence of physical symptoms of AD with biochemical evidence of low circulating T. Common symptoms are a reduction of *libido* and erectile strength, fatigue, reduced physical strength and endurance as well as sometimes impaired cognitive function and mood disturbances (50).

### Primary male hypogonadism

Primary male hypogonadism (HG) is defined by low serum T in combination with increased luteinizing hormone (LH). Normal T and high LH levels characterize compensated hypogonadism, which represents impaired testicular function that is rescued by increased LH stimulation. Compensated hypogonadism is subclinical, but increases the likelihood to progress to overt AD when compared to the eugonadal state (51). Congenital primary HG can be caused by gonadal dysgenesis and cryptorchidism (52), as well as by autosomal or sex chromosome aneuploidies like in Klinefelter syndrome (53, 54).

### Secondary male hypogonadism

Secondary HG, or hypogonadotropic HG, is defined by low T and reduced gonadotrophin secretion due to impaired hypothalamic–pituitary stimulation of testicular androgen synthesis. The overwhelming majority of such cases are caused by tumours of the hypothalamo–pituitary area. Congenital hypogonadotropic hypogonadism may be observed in the context of multiple pituitary hormone deficiencies in conditions such as septo-optic dysplasia, but more commonly is associated with isolated gonadotrophin deficiency as observed in Kallmann syndrome, which may be associated with anosmia and cranio-facial abnormalities (55).

### Acquired male hypogonadism

Acquired HG may be caused by lesions or tumours of the central nervous system or testis, radio- and chemotherapy, pharmacological treatment, chronic illness, poor health and obesity (2). Surgical or pharmacological androgen deprivation therapy is an established treatment option for both metastatic hormone-naive and castration-resistant prostate cancer (56).

Ageing affects the hypothalamic–pituitary–gonadal (HPG) axis and can lead to late-onset AD, which is defined as low T levels if any form of classical causes of AD can be excluded (57). Ageing can result in gradual development of testicular failure due to a decreased number and response to LH of Leydig cells, and in reduced hypothalamic–pituitary signalling (58, 59). This manifests in an age-related decline of T levels of around 0.1 nmol/L per year starting during the third decade of life (60).

Male AD can also be induced by obesity (61). Obesity significantly increases the age-related T decline and is associated with disordered gonadotrophin release (60). Conversely, weight loss can reverse obesity-associated hypogonadism (62). The concept of a hypogonadal–obesity–adipokine cycle is a proposed mechanism behind this association (50, 63, 64): Obesity has been suggested to lead to enhanced aromatisation of androgens to oestrogens by aromatase (CYP19A1, Fig. 2) in adipose tissue, thereby reducing the level of active androgens. Oestrogens may suppress the HPG axis, which reduces gonadal T synthesis (65). Treatment of obese men with the CYP19A1 inhibitor letrozole normalises T levels (66). Additionally, elevated levels of adipocyte-derived inflammatory cytokines (67, 68) have been shown to inhibit the HPG axis in healthy men and a contribution of leptin excess to the reduction of androgens in obesity has been suggested (69).

## Androgens and metabolic health in transgender patients

Replacement and blockade of sex hormones underpin the principle of gender reassiginment, both before and after gonadectomy where appropriate, thereby enabling the development of secondary sex characteristics of the desired gender. Circulating sex hormones should be maintained in the upper-normal physiological reference range for the desired gender (70, 71). However, metabolic consequences for androgen deprivation and replacement therapy may be observed in both male-to-female and female-to-male gender reassignment patients (72).

### Female-to-male gender reassignment

For female-to-male gender reassignment, T is administered both before and after genital reconstruction surgery, aiming to induce virilisation and suppress female secondary sex characteristics. Target serum T levels are generally between 12 and 24 nmol/L (70). Long-term T administration for female-to-male gender transition increases total lean mass (73) and visceral fat mass, while reducing subcutaneous fat mass (74); BMI may be increased (75, 76). Surrogate cardiovascular risk factors have been reported to be increased by T administration, including arterial stiffness, blood pressure (77) and dyslipidaemia (78). Female-to-male transgender patients also show an increased prevalence of T2DM compared to female control populations (79, 80). Despite presenting with PCOS symptoms, female-to-male transgender patients taking T show ovarian hyperplasia, but no polycystic ovarian morphology (81) further supporting that AE and not ovarian dysfunction drives the metabolic phenotype in PCOS.

### Male-to-female gender reassignment

Oral or transdermal oestrogen supplementation is the primary treatment for feminization of male-to-female transgender patients, both before and after orchidectomy; anti-androgen treatment is frequently co-prescribed in the pre-gonadectomy stage (71). Serum T levels <1.9 nM are recommended (71). Delineating the specific effects of androgen deprivation therapy in this patient population is clouded by co-administration of relatively large doses of oestrogen. Male-to-female transgender patients on combined estrogen and anti-androgen treatment develop an adverse lipid profile (76, 78) with reduced muscle mass and total lean mass percentage, but increased subcutaneous and visceral fat mass (73, 74). Prevalence rates of T2DM, thrombo-embolic disease and cerebrovascular disease compared to control men appear to be increased (79).

## The role of androgens in metabolic target tissues

In addition to their central role in the development and maintenance of male and female reproduction and sex drive, androgens exert key effects on metabolic target tissues. These include adipose tissue and skeletal muscle, compartments crucially involved in maintaining systemic glucose and lipid homeostasis.

### Androgens, adipose tissue and lipid metabolism

There is a clear sexual dimorphism in patterns of body fat distribution, with women having a higher percentage of body fat than men, while men have greater total lean mass. In women, body fat is distributed in a gynaecoid manner, with less visceral but more subcutaneous (SC) fat; men have a predominant android fat distribution, with more visceral and less SC adipose tissue (82, 83, 84). Adipose tissue expansion is a consequence of both hyperplasia (adipogenesis), which is driven by proliferation of preadipocytes and their differentiation into adipocytes, and hypertrophy, which is driven by accumulation of lipid in differentiated adipocytes; both processes are major determinants of metabolic dysfunction (85).

Androgens impair adipogenesis by inhibiting proliferation and differentiation of mesenchymal stem cells and preadipocytes (86). DHT and T have inhibitory effects on multipotent stem cell commitment to the preadipocyte lineage, and adipocyte differentiation in both sexes (87, 88). In addition, DHEA, but not DHEAS, has been shown to inhibit proliferation and differentiation of a human SC preadipocyte cell line and to enhance basal glucose uptake (89). Klöting *et al*. hypothesise that an impairment of adipocyte proliferation and differentiation may lead to adipocyte hypertrophy as a compensatory mechanism to increase adipose tissue mass, which could induce adipocyte dysfunction manifested in IR, intracellular stress and inflammation (90).

Hypertrophic, dysfunctional adipocytes induce a proinflammatory, diabetogenic and atherogenic serum profile (90). However, comprehensive human *in vivo* studies evaluating the direct effects of androgens on the secretion of cytokines by adipose tissue are lacking. Incubation with active androgens in primary cultures of human abdominal SC and omental adipocytes from male and female donors (88) showed no significant effect on adiponectin secretion, which has systemic insulin-sensitizing effects. However, women with PCOS have lower levels of adiponectin than healthy controls (91), and hypogonadal men show higher adiponectin than eugonadal men (92) suggesting a potential role for androgens in adiponectin secretion.

Androgens may modulate the balance between lipid catabolism and lipid accumulation. However, studies to date have shown conflicting results. T and its precursor DHEAS have been shown to stimulate lipolysis in humans in a sex- and depot-specific manner (93, 94, 95, 96, 97). Conversely, Corton *et al*. have compared the expression profile of omental adipocytes in obese women with AE to the profile of obese controls with normal androgens revealing hints at enhanced lipogenesis (98, 99) and thus at a possible role of androgens in promoting adipose lipid accumulation.

**Figure 3:**
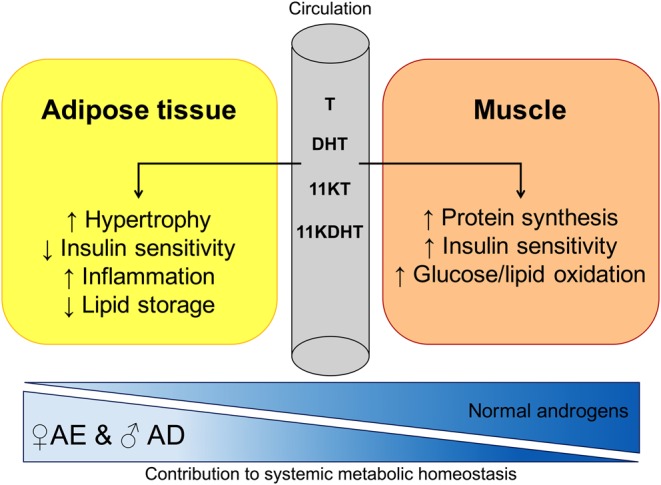
Differential effects of androgens on adipose tissue and skeletal muscle and implications for global metabolism. Androgens may exert pro-lipogenic effects on adipose tissue, resulting in fat mass expansion. At higher concentrations, as observed in the healthy male range, net anabolic effects on increasing skeletal muscle bulk predominate. However, with circulating androgen levels in the range of female androgen excess and male androgen deficiency, a loss of muscle mass and an increase in abdominal obesity drive the systemic phenotype, and give rise to metabolic and cardiovascular disease. Testosterone (T), dihydrotestosterone (DHT), 11-keto-testosterone (11KT), 11-keto-dihydrotestosterone (11KDHT).

Androgens also exert direct and indirect effects on adipose insulin sensitivity. T directly induces IR in female SC adipocytes *in vitro*, and inhibits insulin-stimulated glucose uptake by impairing phosphorylation of protein kinase C via an AR-mediated mechanism (100). The adipose gene expression studies by Corton *et al*. comparing adipocytes from women with and without PCOS show distinct changes in several biological pathways, including oxidative stress, inflammation and lipid metabolism (98). Effects of androgens on adipose tissue are summarised in Fig. 3.

### Androgens, skeletal muscle and insulin sensitivity

Androgens enhance the differentiation of stem cells to myotubes, as well as skeletal muscle protein synthesis, lipid oxidation, insulin sensitivity and glucose usage and mitochondrial function (64, 101) (Fig. 3). The intake of T in combination with non-aromatisable synthetic androgens increases the number of myonuclei, resulting from fusion with satellite cells and promoting muscular growth, and proportion of central nuclei indicative of muscle repair in human skeletal muscle of athletes compared to non-steroid users (102). T stimulates the proliferation and differentiation of satellite cells (103), which can subsequently fuse with the adjacent myofiber. Additionally, androgens induce myogenic differentiation and inhibit adipogenesis of pluripotent mesenchymal stem cells via an AR-dependent pathway (104). Healthy men receiving intramuscular T injections exhibit increases in skeletal muscle protein synthesis (105). Intramuscular T replacement in hypogonadal men confirms the effect of T in reducing protein oxidation (106). In men, T correlates with genetic and functional markers of mitochondrial function in skeletal muscle (107), consistent with findings reporting a positive effect of T on mitochondrial biogenesis and maintenance in skeletal muscle of mice (108, 109).

Incubation of primary human muscle cells with T leads to an upregulation of insulin receptor substrate-2 (110). In cultured rat muscle cells, the addition of T and DHEA enhances GLUT4 expression and translocation to the plasma membrane as well as intracellular insulin signalling (111). T and DHEA stimulate the activity of phosphofructokinase, the key regulatory enzyme of glycolysis, and hexokinase, which phosphorylates free glucose, thereby impairing its release from the cell and channelling it into the pathways of glycolysis, glycogen synthesis or the pentose phosphate pathway (111). T administration leads to increases in muscle glycogen levels in rat (112) due to reduced glycogen breakdown (113). In summary, current evidence suggests that androgens stimulate insulin sensitivity and glucose utilisation in skeletal muscle cells, in both men and women but with sex-specific gradual differences, hinting at a stronger effect in females (110).

## Insulin resistance, type 2 diabetes mellitus and androgen status in men and women

Insulin resistance is defined as the impaired systemic metabolic response to insulin, which includes glucose uptake and metabolism, suppression of lipolysis and promotion of lipogenesis, as well as protein and glycogen synthesis (114). IR is accompanied by compensatory hyperinsulinemia, leading to an exaggerated insulin response in normally less responsive tissues, as well as disturbances in hepatic and adipose lipid metabolism. Frank hyperglycaemia occurs after decompensation of the exaggerated pancreatic beta-cell response to systemic insulin resistance. Studies selected from those discussed in the following sections are summarised in Table 1.

**Table 1:** Selected studies highlighting the effects of androgens on metabolic dysfunction in men and women.

**Metabolic outcomes/Sex**	Study design	Parameters assessed: Main outcome	Reference
Body composition			
M	139 PCOS grouped according to combination of PCO, AO and AE	BMI: No differenceWHR: ↑ in groups with AE, highest in PCO + AO + AE	(135)
M	60 PCOS (biochemical and/or clinical AE) vs 60 controls matched for age, race, BMI	WHR: ↑ in PCOS% body fat: ↑ in PCOSLean mass: No differenceFat–lean–mass ratio: ↑ in PCOS	(131)
F	130 nonsmoking men, age 21–70	Body fat mass, % body fat, WC, vic adiposity: Negatively associated with T and DHEAS	(121)
M/F	17 female-to-male transsexuals on T supplementation followed over 1 year	T levels: ↑ to supraphysiol levelsBody fat distribution: ↓ SC, ↑ vis fatTG: ↑HDL: ↓	(206)
IR and T2DM			
M	86 PCOS grouped according to severity of AE vs 43 controls (matched for age and BMI)	T and A4, IGT, fasting insulin, HOMA-IR: ↑ with severity of AE	(25)
M	15 PCOS on resveratrol treatment vs 15 PCOS placebo controls	T, DHEAS: ↓ by resveratrolFasting insulin: ↓ by resveratrolISI: ↑ by resveratrol	(119)
F	1413 men, age ≥20	T levels, Prevalence of diabetes: Negative association: Free T, bioavailable T and diabetes persisting upon exclusion of men with abnormally low T	(207)
F	156 obese, hypogonadal, diabetic men on T therapy followed over 6 years	Fasting insulin, glycated Hb, WC, weight, blood pressure: ↓Lipid profile: Ameliorated	(128)
NAFLD			
M	Prospective cross-sectional study involving 314 PCOS women and 74 controls	Various liver fibrosis scores, HOMA-IR, HOMA-β, QUICKI: Indices of hepatic steatosis were all significantly higher in the PCOS than the control group, as well as in PCOS women with rather than without metabolic syndrome	(151)
M	Prospective case control study with 29 PCOS women and 29 controls	HOMA-IR, MRI liver, MRS: Differences in liver fat remained apparent after adjusting for differences in obesity and insulin resistance	(152)
F	Retrospective cross-sectional observation study of 495 healthy Korean men	Serum testosterone, BMI, HDL, TG: Low serum T was associated with higher risk of NAFLD independent of vis fat and IR	(154)
F	Prospective cohort study of 55 men with chronic spinal cord injury	Serum T, ultrasonography liver, HOMA-IR: Low T was independently associated with NAFLD	(208)
F	Cross-sectional population-based study of 1912 men	Serum T, serum DHEAS, ultrasonography liver: Hepatic steatosis was associated with low T and high DHEAS	(155)
Dyslipidaemia and CVR			
M	PCOS on hypocaloric diet and flutamid (17) or placebo (19) treatment	A4, DHEAS: ↓ secondary to flutamideVis/SC fat TG, cholesterol, LDL: ↓HDL: Trend for ↑	(141)
M	40 PCOS vs 20 normoandrogenic controls	CIMT: ↑ in PCOS; Correlation with total T, free T, A4 and DHEAS	(164)
M	2301 PCOS (evidence of AE in 88%) followed over 20 years	T2DM, MI, angina, HF, stroke, CV related death: ↑ age-specific prevalence of T2DM, MI, angina compared to local male population	(172)
F	255 hypogonadal men receiving T therapy for 60 months	T levels: ↑ to physiological levelsTG, LDL, blood pressure, glucose, glycated HbA, CRP, liver enzymes: ↓HDL: ↑	(209)
F	4736 men with low T supplemented to persistently low, normal or high T for 3 years	MACE (stroke, MI, death): ↓ in normal T compared to persistenly low T; ↑ stroke risk for high T compared to normal T	(198)

A4, androstenedione; BMI, body mass index; CIMT, carotid intima-media thickness; CRP, C-reactive protein; CV, cardiovascular; CVR, cardiovascular risk; DHEA, dehydroepiandrosterone; DHEAS, dihydroepiandrosterone sulfate; HbA, haemoglobin A; HDL, high density lipoprotein; HF, heart failure; HOMA-β, homeostatic model assessment of β-cell function; HOMA-IR, homeostatic model assessment of insulin resistance; IR, insulin resistance; ISI, insulin sensitivity index; LDL, low density lipoprotein; MACE, major adverse cardiovascular event; MI, myocardial infarct; MRI, magnetic resonance imaging; MRS, magnetic resonance spectroscopy; NAFLD, non-alcoholic fatty liver disease; QUICKI, quantitative insulin sensitivity check index; Ref, reference; SC, subcutaneous; T, testosterone; T2DM, type 2 diabetes mellitus; TG, triglycerids; vis, visceral; WC, waist circumference; WHR, waist–hip ratio.

### Female androgen excess and insulin resistance

The presence of AE in PCOS is closely correlated with insulin resistance. Women with PCOS show a trend to progress from normal glucose tolerance to impaired glucose tolerance (IGT) and to T2DM, and obesity significantly increases this risk (115). Both obese and non-obese PCOS women with AE show a higher prevalence of IGT and T2DM than controls, but obesity deteriorates the diabetic phenotype (116, 117). Conversely, T levels are significantly higher in women with T2DM even after adjustment for age, race, diabetes diagnosis criteria, BMI and waist-to-hip ratio; consequently, AE in women has been suggested as risk factor for T2DM (118). When grouping PCOS patients according to severity of AE, insulin sensitivity decreases and risk of overt hyperglycaemia increases across a spectrum or increasing androgen burden (25). Lowering circulating androgen burden in PCOS by treatment with resveratrol has been shown to reduce fasting insulin and to improve the insulin sensitivity index (119). *In vitro* studies demonstrated selective inhibition of proliferation and androgen production of rat ovarian theca-interstitial cells by resveratrol (120).

### Male androgen deficiency and insulin resistance

In men, T levels are positively associated with insulin sensitivity (107, 121) and even in men with an established diagnosis of T2DM, low T is independently associated with IR (122). A meta-analysis correlating significantly lower T levels in men with T2DM also found the inverse association in women, with higher T levels predicting hyperglycaemia (118). The significance of this correlation is attenuated, but still significant, after adjustment for IR (123, 124). An increase in the prevalence of subnormal T has been found in diabetic men when compared to BMI-matched controls (125). This identifies low T levels as a risk factor for T2DM, independent of obesity. Men with prostate cancer on androgen deprivation therapy have higher BMI, fasting glucose, leptin levels and HOMA-IR compared to healthy controls, with significant negative correlations between total and free T and IR parameters observed (126). Androgen replacement therapy improves insulin sensitivity and diabetes in obese and non-obese hypogonadal men (127, 128).

## Body composition and impact of androgen status in men and women

Similar to the gender-specific effects observed for androgen effects on systemic IR, there are sexually dimorphic effects of androgens on body composition.

### Female androgen excess and body composition

PCOS women with clinical and/or biochemical evidence of AE show a higher prevalence of obesity than the general female population (4) and an increased global adiposity compared to control cohorts (129). In a detailed study comparing hyperandrogenic PCOS women, healthy women and men, Borruel *et al*. demonstrated increased amounts of visceral fat depots in women with PCOS in addition to the increased global adiposity (130). They have an increased body fat mass resulting in a higher body fat-to-lean mass ratio, which is positively associated with metabolic risk (131, 132). For women with and without PCOS, BMI correlates with the FAI and systemic 5α-reductase activity (25) and body weight, waist circumference and waist-to-hip ratio are higher in the presence of AE in PCOS (133, 134, 135). Women presenting with isolated hirsutism show significantly higher increases in BMI during early adulthood than controls (136). A recent study found a significant positive correlation between circulating androgens with body fat mass and obesity in pre-pubertal and pubertal girls (137). Studies on PCOS women with AE describe an increased lean mass correlating with serum T and A4 (138, 139), with a shift in fat distribution from a gynaecoid to an android pattern (132). The treatment of PCOS women on a hypocaloric diet with the anti-androgen flutamide decreases androgen levels and the visceral-to-SC fat ratio (140, 141).

### Male androgen deficiency and body composition

In comparison to women, circulating androgens in men correlate inversely with BMI and visceral adiposity. Cross-sectional studies analysing age-advanced men, men across different ages and obese vs non-obese men consistently support the association between low T and increased fat mass compared to eugonadal controls (107, 142, 143). BMI negatively correlates with total and free T (142, 144), and waist circumference is negatively associated with total T in men (142). Although age is associated with decreased androgen levels (143, 144), negative associations between T and total body fat mass, body fat percentage, waist circumference and visceral adipose tissue are maintained after adjustment for age (121). T administration in men reduces accumulation of visceral and retroperitoneal fat compared to controls, but not in SC depots; hypogonadal men also have increased visceral fat mass (145). Lean body mass is lower in hypogonadal men compared to eugonadal controls (146, 147). T replacement therapy of hypogonadal men leads to increases in lean body mass and reduces vis adiposity in men with and without T2DM (127, 148).

## Non-alcoholic fatty liver disease (NAFLD) and male and female androgen status

NAFLD is an umbrella term encompassing a spectrum of hepatic injury induced by obesity and IR, in the absence of significant alcohol consumption. The NAFLD spectrum ranges from intra-hepatic accumulation of TG or simple steatosis, to diffuse tissue inflammation or non-alcoholic steato-hepatitis (NASH), with a risk of progression to advanced hepatic fibrosis and cirrhosis (149). NAFLD is a major metabolic complications and emerging as the most frequent cause of liver transplantation in the Western world.

### Female androgen excess and NAFLD

Prevalence rates of NAFLD in PCOS appear to be higher than those in BMI-matched individuals from the background population; a recent meta-analysis found that patients with PCOS have an almost 4-fold higher prevalence of NAFLD compared to controls with simple obesity (150). Polyzos and colleagues reported a significantly higher prevalence of hepatic steatosis in a large cohort of Mediterranean women with PCOS when compared to the healthy female population. However, they did not find any difference in the prevalence of hepatic fibrosis, which was attributed to younger age of the cohort (151). Jones *et al.* compared several metabolic parameters in PCOS women with and without AE diagnosed according to the Rotterdam criteria and found that liver fat was significantly higher in hyperandrogenic PCOS compared to normoandrogenic PCOS women (diagnosed with PCOS due to PCO + AO), even after adjustment for obesity, IR and visceral and intra-abdominal fat (152). Androgenised female rats fed with a diet rich in advanced glycation end products have been shown to develop deranged hepatic function (153). However, putative causative mechanisms underlying PCOS-related NAFLD remain to be elucidated.

### Male androgen deficiency and NAFLD

Kim *et al.* report that low serum T level was independently associated with NAFLD in Korean men despite adjusting for traditional risk factors such as visceral adiposity and IR (154). A large observational study of German men also reported an inverse association between serum T and hepatic steatosis (155). Although indirect mechanisms, such as increased visceral adiposity in the context of hypogonadism, were initially hypothesised, recent studies have underpinned a direct role for androgens on liver metabolism. Liver-specific male AR knock-out mice develop hepatic steatosis and IR with a high fat diet. This appears to be due to activation and upregulation of sterol regulatory element binding protein-1c and acetyl coA carboxylase, coupled with a reduction in peroxisome proliferator-activated receptor-alpha and malonyl coA decarboxylase expression. This results in increased malonyl co-A, a substrate for *de novo* lipogenesis and negative regulator of carnitine palmitoyltransferase 1, which is a major regulator of beta-oxidation (156). Mirroring female AE, however, excessive androgen replacement and supraphysiological serum androgens may also adversely impact on risk of NAFLD in men. Synthetic anabolic steroid use has been linked to hepatic steatosis in men (157), again suggesting the presence of a relatively narrow physiological window outside of which adverse metabolic consequences may arise.

## Cardiovascular risk and male and female androgen status

### Female androgen excess and cardiovascular risk

According to a recent meta-analysis, AE in PCOS is associated with higher total cholesterol and lower HDL levels, but does not affect TG and LDL levels (158). Studying the direct associations between AE and dyslipidaemia is confounded by co-existent obesity and IR in most PCOS studies. Nevertheless, treatment with the anti-androgen flutamide improves the dyslipidaemic phenotype in both obese and non-obese women and leads to decreases in T and A4 levels probably secondary to normalisation of ovulation and gonadotrophin secretion (140, 141, 159). Despite large inter-study heterogeneity, profiles of circulating markers for systemic inflammation, oxidative stress and coagulation disorders appear to be altered in PCOS, indicating an increased CVR (91, 160, 161, 162, 163).

Luque-Ramirez *et al*. comparing hyperandrogenic PCOS with non-hyperandrogenic women showed an increased mean carotid intimal media thickness (CIMT) in PCOS, independent of obesity, and indentified total T and A4 as major determinants of CIMT (164). Women with PCOS and AE also exhibit microvascular dysfunction due to impaired vasodilation (165, 166). Data on long-term cardiovascular events in PCOS are inconsistent. Some studies conclude that there is no increased risk for large vessel disease (167), abdominal aortic plaque (168), myocardial infarction (MI) or stroke (169, 170). Others describe increases in the prevalence of hypertension (168, 170) and cerebrovascular disease (171), in the age-specific prevalence of MI and angina (172) and in the risk of coronary heart disease and stroke, even after adjustment for BMI (173). General and cause-specific mortality and age at death may not be significantly higher in PCOS women than the background population (167, 169, 170, 174).

### Male androgen deficiency and cardiovascular risk

In men, low T levels are associated with a dyslipidaemic profile. An inverse relation between T and TG, total cholesterol and LDL as well as a positive correlation of total and free T with HDL (175, 176, 177, 178, 179, 180) was described. ADT for the treatment of PCa also induces dyslipidaemia (181, 182, 183), while T replacement therapy in hypogonadal men exhibits beneficial effects on the lipid profile (127, 184, 185). An inverse correlation exists between serum T and high-sensitive C-reactive protein in normal ageing (186) and hypogonadal (187) men, and T replacement has been shown to shift the cytokine balance towards a state of reduced inflammation (184, 188).

Increased arterial stiffness has been reported in hypogonadal men compared to age- and weight-matched controls, which can be rapidly but incompletely rescued by T supplementation (189). Men with coronary artery disease present with lower T levels (190, 191) and its severity is negatively correlated with T levels (190, 192, 193). Male AD is associated with a higher risk of all-cause mortality (194, 195), and an inverse correlation exists between T levels and prospective mortality due to all causes, cardiovascular disease and cancer (196). We found that men with gonadotrophin deficiency after the treatment of non-functioning pituitary adenomas had increased mortality compared to their eugonadal counterparts (197). Supplementing men with initially low T levels to normal T levels reduces the rate of stroke, MI or death compared to subjects with persistently low T (198, 199).

## Conclusions

Androgens play a major role in human metabolic health and disease. Female androgen excess and male androgen deficiency exhibit overlapping metabolic phenotypes, highlighting the complexity of the role of androgens in metabolism (Fig. 1). Effects of androgens on adipose tissue and muscle may largely be governed by circulating serum and tissue-specific concentrations, with a narrow physiological window in both sexes, outside of which disturbances in metabolism and body composition are observed. In healthy women, low androgen concentrations and elevated oestrogens lead to predominant gynaecoid fat distribution and reduced metabolic risk; at circulating androgen levels observed in severe female AE and male AD, preferential accumulation of central and visceral adiposity is observed, while at higher androgen concentrations seen in healthy men, this effect is dissipated by increasing lean body mass, muscle bulk and reducing fat mass (Fig. 3). Further human-based studies, including *in vitro*, *in vivo* and epidemiological studies appropriately taking into account sex differences, are required to understand and dissect these complex associations.

## Declaration of interest

The authors declare that there is no conflict of interest that could be perceived as prejudicing the impartiality of this review.

## Funding

This work was supported by the Wellcome Trust (Clinical Research Training Fellowship 099909, to M O R, and Project Grant 092283, to W A).
